# Baseline anemia and its associated factors among adult cancer patients at Northwest Amhara Regional State Referral Hospitals, Northwest Ethiopia, 2021

**DOI:** 10.3389/fonc.2024.1390052

**Published:** 2024-06-19

**Authors:** Yilkal Abebaw Wassie, Alebachew Ferede Zegeye, Deresse Abebe Gebrehana, Sintayehu Simie Tsega, Getasew Kibralew, Setegn Fentahun, Abebaw Setegn, Girum Nakie

**Affiliations:** ^1^ Department of Medical Nursing, School of Nursing, College of Medicine and Health Sciences, University of Gondar, Gondar, Ethiopia; ^2^ Department of Internal Medicine, School of Medicine, College of Medicine and Health Sciences, University of Gondar, Gondar, Ethiopia; ^3^ Department of Psychiatry, College of Medicine and Health Science, University of Gondar, Gondar, Ethiopia; ^4^ Department of Medical Parasitology, School of Biomedical and Laboratory Sciences, College of Medicine and Health Science, University of Gondar, Gondar, Ethiopia

**Keywords:** anemia, prevalence, cancer, Gondar, Ethiopia

## Abstract

**Introduction:**

Currently, the problem of cancer has been increasing around the world, predominantly in middle- and low-income countries. Anemia, a major and often overwhelming health burden for cancer patients, significantly distorts their quality of life. It is well-established that the length of treatment increases the frequency of anemia, with hematological malignancies experiencing nearly double the rate compared to solid tumors. Despite this established knowledge, data on the prevalence of anemia among cancer patients in Ethiopia remains scarce, according to the investigators.

**Objective:**

This study aimed to assess the prevalence of baseline anemia and associated factors among adult cancer patients at Northwest Amhara Comprehensive Specialized Hospitals, oncology treatment units, Northwest Ethiopia, in 2021.

**Methods:**

This study employed an institutional-based cross-sectional design and was conducted in Northwest Amhara Comprehensive Specialized Hospitals. A systematic random sampling technique was used to select 315 participants. The data were collected using interviewer-administered questionnaires and chart reviews of existing medical records using a structured and pretested questionnaire format. The data were entered into Epi. Data version 4.6 and analyzed using Stata version 14.0. Bivariable and multivariable logistic regression were carried out to identify factors associated with anemia. Adjusted odds ratios with a 95% confidence interval and variables with a *p*-value of < 0.05 were considered significantly associated with anemia.

**Results:**

The prevalence of baseline anemia among adult patients with cancer was found to be 34.84%. Being a woman (AOR = 1.97; 95% CI: 1.00–3.87), being underweight (AOR = 1.96; 95% CI: 1.09–3.52), and having stage III cancer (AOR = 2.35; 95% CI: 1.12–3.01) were significantly associated with anemia.

**Conclusion:**

The prevalence of baseline anemia among adult cancer patients was significant. Women, cancer patients with an underweight body mass index, and those diagnosed with clinical-stage III cancer were more likely to have baseline anemia. For health policymakers and healthcare providers, it is better to give special attention to female patients, patients who are underweight, and patients with advanced-stage cancer to reduce the risk of developing the outcome. This would allow for timely intervention to manage anemia and potentially improve treatment tolerance and quality of life for cancer patients.

## Introduction

Approximately one-sixth of all fatalities globally are caused by cancer, making it the most prevalent public health issue. In 2020 alone, nearly 9.6 million deaths were reported worldwide, which may be attributed to something (1). Cancer patients are particularly vulnerable to anemia, which is a major and debilitating health condition that can significantly impair their quality of life. A pathological decrease or reduction in hemoglobin (Hb) concentration, hematocrit, or the number of red blood cells per liter under the reference value for a healthy individual of similar age, gender, and race in a congruent environmental setting brought on by various pathophysiological mechanisms is what is meant to be understood (2–4).

Anemia is a global public health concern that affects both advanced and low-income nations, having a significant impact on social and economic advancement as well as human health. Around one-fourth of the world’s population, or 1.93 billion people, suffers from anemia globally. The majority of anemia-related impairments (89%) are seen in low-income nations (5). The magnitude is highest in Central and Western Sub-Saharan Africa, South Asia, Southeast Asia, and East Asia. It occurs at all stages of the life cycle but is more prevalent in patients with chronic disease, specifically cancer (6). Cancer is one of the most common conditions associated with anemia, a chronic disease, while anemia is a common complication of cancer (7). Most cancer patients, especially those who are receiving therapy, suffer anemia while their condition is still being treated. However, it most likely happens for unknown causes/means most of the time, following myelosuppressive chemotherapy or radiotherapy (8, 9).

Different articles announced that the magnitude of anemia varies ranging from 30% to 90% of cancer patients for the time of the course of their diseases (10). Numerous factors are recognized to be significant with anemia, as well as sociodemographic factors such as age, gender, ethnicity, locality, and marital status; nutritional factors, which are determined by overweight, obesity, and an imbalanced diet; lifestyle factors such as physical activity; and psychological factors such as depression and anxiety (7, 11).

Chemotherapy is one of the most essential causes of anemia in cancer patients, and the association between the dose and duration of chemotherapy with anemia is well known. The length of treatment increases the frequency of anemia (12). Before the administration of chemotherapy, every patient has an electrolyte assessment, including the measurement of hemoglobin. The frequency of anemia in hematological malignancies is nearly double that of solid tumors (13). This anemia can severly impact a patient’s mental, physical, and social development in both the short and long term; it can lead to problems with the immune system, hinder motor and cognitive growth, cause poor performance in school, and reduce work productivity throughout the patient’s life, thereby reducing earning capacities and adversely affecting national economic development (14).

Despite the challenges, chemotherapy may still be justified in patients with anemia by considering several factors, including cancer type and stage (15). The potential benefits of chemotherapy for a particular cancer and its stage outweigh the risks associated with anemia (16). Severity of anemia: the degree of anemia can influence the decision (17). Milder anemia might be manageable with supportive measures. Patient’s overall health: a patient’s overall health and ability to tolerate treatment are crucial considerations (18).

Generally, anemia can also distort many features of quality of life (QOL) and result in functional deficits (3, 7, 8). However, there are little data known about anemia among patients with cancer in African countries, including Ethiopia. Therefore, the purpose of this study was to determine the magnitude of baseline anemia and its associated factors among patients living with cancer.

## The objective of the study

### General objective

The purpose of this study is to assess the prevalence of baseline anemia and associated factors among adult cancer patients at Northwest Amhara Comprehensive Specialized Hospitals, oncology treatment units, Northwest Ethiopia, in 2021.

### Specific objectives

This study, conducted in 2021 at Northwest Amhara Comprehensive Specialized Hospitals, oncology treatment units, Northwest Ethiopia, aimed to determine the prevalence of baseline anemia among adult cancer patients and to identify factors associated with baseline anemia in these patients.

## Materials and methods

### Study design and period

An institutional-based cross-sectional study was adopted for this research, which was conducted from 15 March to 15 May 2021.

### Study settings

The study was conducted at Northwest Amhara Comprehensive Specialized Hospitals. There are a total of eight comprehensive specialized hospitals in the Amhara Region, of which five—Debre Markos, Felege Hiwot, Tibebe Gion, Debre Tabor, and University of Gondar—are found in the Northwest of Amhara. Each comprehensive specialized hospital serves 3.5–5 million people (19). Of the five referral hospitals, two [University of Gondar Comprehensive Specialized Referral Hospital (UoGCSH) and Felege Hiwot Comprehensive Specialized Hospital (FHRH)] have oncology treatment units. Those two referral hospitals are located in the Amhara Regional State, Northwest Ethiopia, 738 km and 565 km away from the capital city of Ethiopia, Addis Ababa, respectively. There are a total of 980 cancer patients in the two referral hospitals. The Oncology Treatment Unit of UoGCSH was established in 2014 G.C. and currently has 500 cancer patients and 17 beds for the management of cancer patients. Whereas the oncology treatment unit of FHCSH was established in 2016 G.C. and has 480 cancer patients, it currently has 18 beds for inpatient treatment of cancer patients. A 1-month average number of cancer patients who had follow-up treatments in UoGCSH and FHCSH were 250 and 240, respectively.

### Study participants

All adult cancer patients who were ≥ 18 years old and patients who attended Northwest Amhara Comprehensive Specialized Hospitals and oncology treatment units during the data collection period were included in the study. Those adult cancer patients with transferred-in record information were excluded because these charts may lack baseline information.

### Sample size determination and sampling procedure

The sample size was calculated using the single population proportion formula, considering the following: a 95% confidence interval (CI), a 23% proportion of anemia from the previous study conducted in Addis Ababa (20), and a 5% margin of error. The final sample size was 315, considering a 10% nonrespondent rate. A systematic random sampling method was employed to select study participants in the study area by using the order of the cases. The sample size was proportionally allocated for each selected hospital based on their number of cancer patients and based on the report obtained from the oncology treatment units. The first participant to be included in the study was selected using the lottery method (*K* = *N*/*n*, where *K* is the interval, *N* is the total population, and *n* is the sample size), and then every three participants were interviewed.

### Operational definition

#### Anemia

This study classified participants as anemic or nonanemic based on the WHO criteria: hemoglobin concentration < 13 g/dL for men and < 12 g/dL for women (21).

#### Physical functional status

Physical ability was assessed using the ECOG performance status grade (22). This reflects a patient’s general well-being and his/her ability to perform activities of daily life.

#### Body mass index

In this study, the calculation of body mass index (BMI) involves dividing the weight of an individual in kilograms by their height in square meters (23).

### Data collection instruments and procedures

The data were collected by using interviewer-administered and chart-reviewed structured pretested questionnaires that are adapted from a questionnaire developed by a previous study, which contains four sections. The first section contains eight questions regarding sociodemographic characteristics of the study participants; the second section contains seven questions related to the clinical characteristics of a patient; the third section includes the nutritional status of a patient; and the fourth section is related to the behavioral factors of a patient with cancer. The data were collected by four BSc nurses who have working experience in the oncology treatment unit. Adult cancer patients were identified during their follow-up visits to the oncology unit. Patients who met the inclusion criteria were then interviewed after obtaining their consent to participate in the study.

### Data processing and analysis

After data collection, the collected data were cleaned and checked for completeness. Data were entered using Epi Data version 4.6 after being coded and analyzed using Stata version 14.0. Descriptive statistics were used in the analysis of medians and frequencies, and percentages were computed for all variables. Data were presented in tables. The association between dependent and independent variables was assessed using a binary logistic regression analysis model to estimate the strength of association using odds ratios (OR). All variables associated with baseline anemia with a *p*-value less of than 0.25 in the bivariable analysis were further analyzed using multivariable analyses to control potential confounding factors. Variables with a *p*-value of less than 0.05 were declared to be associated with baseline anemia. Multicollinearity and model goodness-of-fit tests were checked by using variance inflation factors (VIF = 1.05–4.17) and Hosmer and Lemeshow goodness-of-fit test (*p* = 0.91), respectively.

### Data quality control

A pretest was done on 5% of the total sample size to make sure whether the questionnaire was appropriate and to ensure its validity in the study population before the actual data collection time. After the pretest, training was given to all data collectors and supervisors on the purpose of the study, how to get informed consent, and the technique of selecting the study participants from each oncology treatment unit. Supervision was conducted by the supervisors and the principal investigator. All questionnaires were translated into local languages (Amharic) before data collection. Consistency was checked by a backtranslation by another expert fluent both in English and in local languages. At the end of each data collection day, the supervisors were checked for the completeness or fulfillment of the questionnaires and the quality of the recorded information.

### Ethical consideration

Ethical clearance was obtained from the School of Nursing research ethical review committee on behalf of the University of Gondar institutional review board. Written permission letters were obtained from hospital managers. Participants were informed about the purpose of the study, and written informed consent was obtained from them. Confidentiality was maintained by omitting direct personal identifiers on the questionnaire, using code numbers, storing data locked with a password, and not misusing or disclosing their information. Participants were also informed that participation was voluntary and they had the right to withdraw from the study at any stage if they were not comfortable with the investigation. The issues of privacy and confidentiality were strictly maintained.

## Results

### Sociodemographic characteristics

A total of 315 study participants were enrolled in the study, with a response rate of 310 (98.41%). The mean age of the study participants was 45.81, with a standard deviation of ± 10.98 years. Nearly one-third (99; 31.94%) of them were between the ages of 40 and 49 years. A bit less than three-fourths (218; 70.32%) were women, and the majority (284; 91.61%) were followers of Orthodox Christianity. Most of them (292; 94.19%) were Amhara by ethnicity, and 209 (67.42%) were rural dwellers (**Table 1**).

**Table 1:** Sociodemographic characteristics of adult patients with cancer in Northwest Amhara Comprehensive Specialized Hospitals, oncology treatment units, 2021 (*n* = 310).

Variables	Category	Frequency (n)	Percent (%)
Age	< 30	37	11.92
	30–39	41	13.23
	40–49	99	31.94
	50–59	96	30.97
	> 60	37	11.94
Sex	Male	92	29.68
	Female	218	70.32
Marital status	Single	22	7.10
	Married	219	70.65
	Divorced	38	12.25
	Widowed	31	10.00
Religion	Orthodox	284	91.61
	Muslim	20	6.45
	Protestant	6	1.94
Ethnicity	Amhara	292	94.19
	Agew	16	5.16
	Others^a^	2	0.65
Residence	Urban	101	32.58
	Rural	209	67.42
Educational status	No formal education	168	54.19
	Primary school	86	27.75
	Secondary school	31	10.10
	Collage and above	25	8.06
Occupation	Employed	85	27.42
	Farmer	29	9.35
	Merchant	48	15.48
	Housewife	117	37.74
	Unemployed	16	5.16
	Others^a^	15	4.84

a Kimant, Tigre.

### Clinical-related characteristics of patients

Of the total (310) cancer patients who participated in the study, nearly one-fourth (74; 23.87%) had gastrointestinal cancer; three-fourths (224; 42.26%) presented at a late stage of cancer (stages III and IV); and nearly half (143; 46.13%) had comorbidities, of which hypertension accounted for 18.71% (**Table 2**).

**Table 2:** Clinical-related characteristics of adult cancer patients in Northwest Amhara Comprehensive Specialized Hospitals, oncology treatment units, 2021.

Variables	Category	Frequency (*n*)	Percent (%)
Cancer type	Breast cancer	79	25.48
	GI cancer	74	23.87
	Hematologic cancer	57	18.39
	Gynecological cancer	68	21.94
	Lung cancer	17	5.48
	Prostate cancer	15	4.84
Clinical stage	Stage I	18	5.81
	Stage II	68	21.94
	Stage III	131	42.26
	Stage IV	93	30.0
Duration since diagnosis in months	< 12	218	70.32
	12–24	29	9.35
	25–36	22	7.10
	37–48	16	5.16
	49–60	14	4.52
	> 60	11	3.55
Cycle of chemotherapy	2	51	16.45
	3	52	16.77
	4	70	22.58
	5	82	26.45
	6 and above	55	17.74
ECOG performance	Grade 0	27	8.71
	Grade 1	184	15.01
	Grade 2	95	30.65
	Grade 3	4	4.35
	Grade 4	–	–
Comorbidity	Yes	143	46.13
	No	168	53.87
Type of comorbidity	HIV/AIDS	44	14.24
	Hypertension	58	18.71
	Others^a^	41	13.23
Body mass index (BMI)	Underweight	97	31.29
	Healthy weight	190	61.29
	Overweight	23	7.42
	Obesity	–	–
Substance use	Yes	96	30.97
	No	214	69.03

a Diabetes mellitus, heart failure.

### The overall magnitude of baseline anemia

The prevalence of baseline anemia among patients with cancer in Northwest Amhara Comprehensive Specialized Hospitals, oncology treatment units was found to be 34.84% (95% CI: 29.71–40.34). Of which, women made up 28.38% of the group, with men comprising the remaining 6.46%.

### Factors associated with anemia

A bivariate analysis was carried out to identify factors associated with baseline anemia among adult cancer patients, and gender, age, occupation, BMI, and stage of cancer were significantly associated with anemia. Finally, multivariable logistic regressions were conducted and gender, body mass index, and stage of cancer were significantly associated with anemia among adult cancer patients. Women are nearly two times more likely to develop anemia as compared to men (AOR = 1.97; 95% CI: 1.00–3.87), underweight patients are nearly two times more likely to develop baseline anemia as compared to their counterpart (AOR = 1.96; 95% CI: 1.09–3.52), and patients with stage III cancer are nearly two and a half times more likely to develop anemia as compared to their counterparts (AOR = 2.35; 95% CI: 1.12–3.01) (**Table 3**).

**Table 3:** Bivariable and multivariable logistic regression of factors associated with baseline anemia among adult cancer patients, 2021 (*n* = 310).

Variables	Category	Anemia		COR (95% CI)	AOR (95% CI)	*p*-value
		Yes	No			
Age	< 30	17	20	1	1	
	30–39	18	23	0.92 (0.38–2.25)	1.1 (0.41–2.97)	0.849
	40–49	29	70	0.49 (0.22–1.06)	0.57 (0.24–1.38)	0.215
	50–59	36	60	0.71 (0.33–1.52)	0.97 (0.4–2.38)	0.952
	> 60	8	29	0.32 (0.12–0.90)	0.45 (0.15–1.32)	0.146
Sex	Male	20	72	1	1	
	Female	88	130	2.44 (1.39–4.29)	1.97 (1.00–3.87)	**0.050** ^a^
Occupation	Employed	21	64	1	1	
	Farmer	10	19	1.60 (0.65–3.99)	1.93 (0.44–3.25)	0.730
	Merchant	20	28	2.18 (1.02–4.64)	1.78 (0.77–4.10)	0.175
	Housewife	43	74	1.77 (0.95–3.29)	1.22 (0.60–2.50)	0.572
	Unemployed	9	7	3.92 (1.30–11.82)	1.76 (0.49–6.41)	0.380
	Others	5	10	1.53 (0.47–4.97)	1.30 (0.35–4.66)	0.709
Body mass index	Underweight	74	23	2.01 (1.21–3.64)	1.96 (1.09–3.52)	**0.025** ^a^
	Healthy weight	115	75	1	1	
	Overweight	10	13	1.18 (0.49–2.83)	1.32 (0.51–3.40)	0.559
Clinical stage	Stage I	9	9	1	1	
	Stage II	29	39	0.74 (0.26–211)	0.55 (0.18–1.67)	0.289
	Stage III	97	34	2.85 (1.14–3.05)	2.35 (1.12–3.01)	**0.050** ^a^
	Stage IV	36	57	0.63 (0.23–1.74)	0.50 (0.17–1.47)	0.207

a Others; daily labor; student.

AOR, adjusted odd ratio; COR, crude odd ratio.

Bold value indicated that significantly associated with baseline anemia.

## Discussion

This study revealed the prevalence of baseline anemia and its associated factors among adult cancer patients in Northwest Amhara Referral Hospitals and oncology treatment units. The overall prevalence of baseline anemia among study participants was found to be 34.84% (95% CI: 29.71–40.34). The prevalence of baseline anemia found in this study was in agreement with a study conducted in Europe (39.3%) (24) and Australia (35%) (25). The prevalence of baseline anemia found in this study was higher than the findings of a study conducted in Ethiopia: Addis Ababa (23%) (20). The possible reason for this difference may be due to the difference in sampling method and study time because the prevalence of cancer is increasing every day in the world. Similarly, the result was also higher than the studies done in China (18.98%) (26) and South Korea (14.2%) (27). The discrepancy between our findings and those of these studies might be due to procedural differences, especially sensitivity differences between the different screening apparatuses. It may be due to the fact that classifications of anemia differ somewhat from several references; the World Health Organization explains anemia as an Hb level < 10.9 g/dL (15), whereas the European Organization for Research and Treatment of Cancer (EORTC) and the National Cancer Institute (NCI) (16) describe anemia as a Hb level < 12.0 g/dL (28).

On the contrary, the prevalence of baseline anemia in this study was lower than in studies conducted in India, New York, Thailand, Belgium, Spain, Saudi Arabia, and Israel: 54.7%, 41%, 54.4%, 55.7%, 48.1%, 44.1%, and 53%, respectively (11, 28–33). The difference might be due to differences in the study design. This inconsistency could also be due to differences in the socioeconomic, cultural, and lifestyle differences between those countries. The possible reason may be due to healthcare delivery policy dissimilarities and the country’s right of way to cancer management and prevention in the case of developed countries.

Regarding the associated factors of baseline anemia among adult patients with cancer, women were nearly two times more likely to develop anemia as compared to men. This finding was supported by studies conducted in Belgium (28). This might be due to the physiological effects of women, such as they are more vulnerable to anemia at a reproductive age due to menstrual blood loss and childbearing iron loss. In addition to this, the reason behind this may also be that, due to the present study, most of the respondents were women.

The current study found that patients with advanced stage of cancer were nearly two and a half times more likely to develop anemia as compared to their counterparts. This result was similar to a study conducted in the USA (10), Australia (29), and Norway (34). This might be due to complex communications between tumor cells and the immune system. Overpowering certain inflammatory cytokines results in reduced existence of red blood cells, a clampdown of erythroid progenitor cells, diminished iron utilization, and insufficient erythropoietin production (35, 36). Furthermore, a direct result of the malignancy is that it attacks normal tissues, causing blood loss and marrow infiltration, which prevents the production of red cells, or inflammation, leading to functional iron deficiency.

According to this study, cancer patients with an underweight body mass index were nearly two times more likely to develop baseline anemia as compared to their counterparts. This finding was in line with other studies conducted in Mikki et al. (37). The possible reason might be that, typically, the causes of anemia and underweight (malnutrition) are similar and aggravated by poverty and food insecurity. Food insecurity affects the dietary patterns of cancer patients by compromising the quantity and quality of nutritional consumption, which contributes to the development of anemia (38). This shows that malnutrition has a substantial role in the health of people, and maintaining a healthy weight is essential to improving health in general. Moreover, this may be due to the different dietary intakes, where poor nutritional practices that lead to insufficiencies of iron, folate, or vitamin B12 are also reasons that might contribute to anemia in underweight patients.

## Strength of the study

This research raises awareness about a common problem—anemia—among cancer patients even before treatment begins. This can help with early diagnosis and intervention. Next, it is used to identify factors associated with anemia, which can help healthcare providers predict which patients are more at risk. Early intervention can improve their prognosis and quality of life. Finally, these studies focusing on certain cancers, like cervical cancer, provide valuable data on that specific patient group.

## Limitations of the study

This study appears to have limitations. Firstly, it only included individuals accompanying cancer patients, not the cancer patients themselves. Secondly, it did not assess the level or type of anemia, which are crucial factors for understanding the condition. If the study relies on medical records, the data might not have been collected for the study and could be inaccurate or incomplete. Most variables were self-reported and therefore may be affected by social desirability bias or defensive reactions.

## Conclusion

This study highlights the prevalence of baseline anemia among adult cancer patients. Identifying factors associated with it, like being female, cancer patients with an underweight body mass index, and cancer patients with stage III, could help predict at-risk patients. Early detection of anemia through screening programs for high-risk patients (identified by this study’s factors) could be beneficial. This would allow for timely intervention to manage anemia and potentially improve treatment tolerance and quality of life for cancer patients.

## Data availability statement

The original contributions presented in the study are included in the article/supplementary material. Further inquiries can be directed to the corresponding author.

## Ethics statement

The studies involving humans were approved by School of Nursing research ethical review commute on the behalf of the University of Gondar institutional review board. The studies were conducted in accordance with the local legislation and institutional requirements. Written informed consent for participation was not required from the participants or the participants’ legal guardians/next of kin in accordance with the national legislation and institutional requirements.

## Author contributions

YW: Methodology, Investigation, Funding acquisition, Formal analysis, Data curation, Conceptualization, Writing – review & editing, Writing – original draft, Visualization, Validation, Supervision, Software, Resources, Project administration. AZ: Writing – review & editing, Writing – original draft, Visualization, Validation, Supervision, Software, Resources, Project administration, Methodology, Investigation, Funding acquisition, Formal analysis, Data curation, Conceptualization. DG: Writing – review & editing, Writing – original draft, Visualization, Validation, Supervision, Software, Resources, Project administration, Methodology, Investigation, Funding acquisition, Formal analysis, Data curation, Conceptualization. ST: Writing – review & editing, Writing – original draft, Visualization, Validation, Supervision, Software, Resources, Project administration, Methodology, Investigation, Funding acquisition, Formal analysis, Data curation, Conceptualization. GK: Writing – review & editing, Writing – original draft, Visualization, Validation, Supervision, Software, Resources, Project administration, Methodology, Investigation, Funding acquisition, Formal analysis, Data curation, Conceptualization. SF: Writing – review & editing, Writing – original draft, Visualization, Validation, Supervision, Software, Resources, Project administration, Methodology, Investigation, Funding acquisition, Formal analysis, Data curation, Conceptualization. AS: Writing – review & editing, Writing – original draft, Visualization, Validation, Supervision, Software, Resources, Project administration, Methodology, Investigation, Funding acquisition, Formal analysis, Data curation, Conceptualization. GN: Writing – review & editing, Writing – original draft, Visualization, Validation, Supervision, Software, Resources, Project administration, Methodology, Investigation, Funding acquisition, Formal analysis, Data curation, Conceptualization.
